# Engineering a 3D platform for testis bioengineering: generation and proteomic profiling of decellularized fish testicular scaffolds

**DOI:** 10.3389/fbioe.2025.1631542

**Published:** 2025-07-25

**Authors:** Ivana Felipe Rosa, Beatriz Marques Souza, Lucas Benites Doretto, Maira da Silva Rodrigues, Caroline Nascimento Barquilha, Matheus Naia Fioretto, Luiz Marcos Frediani Portela, José Carlos Souza Vieira, Luis Antonio Justulin, Pedro de Magalhães Padilha, Changwei Shao, Rafael Henrique Nóbrega

**Affiliations:** ^1^Reproductive and Molecular Biology Group, Department of Structural and Functional Biology, Institute of Biosciences, São Paulo State University (UNESP), Botucatu, Brazil; ^2^State Key Laboratory of Mariculture Biobreeding and Sustainable Goods, Yellow Sea Fisheries Research Institute, Chinese Academy of Fishery Sciences, Qingdao, Shandong, China; ^3^Laboratory for Marine Fisheries Science and Food Production Processes, Qingdao Marine Science and Technology Center, Qingdao, Shandong, China; ^4^Laboratory of Extracellular Matrix Biology, Department of Structural and Functional Biology, Institute of Biosciences of Botucatu, Sao Paulo State University (UNESP), Botucatu, Brazil; ^5^Bioanalytics and Metalloproteomics Laboratory, Department of Chemistry, Institute of Biosciences of Botucatu, Sao Paulo State University (UNESP), Botucatu, Brazil

**Keywords:** testicular scaffolds, decellularization, sds, matrix extracellular, Astyanax lacustris

## Abstract

Decellularization represents a robust strategy for generating biologically derived scaffolds that retain the native architecture and biochemical complexity of the extracellular matrix (ECM), thereby providing a conducive microenvironment for germ cell adhesion, proliferation, and differentiation—processes fundamental to the reconstitution of testicular function. While decellularized ECM (dECM) scaffolds have been extensively utilized in mammalian organoid systems for *in vitro* spermatogenesis and fertility-related research, the development of standardized protocols tailored to teleost models remains largely unexplored. In the present study, we established an efficient decellularization protocol for testicular tissue derived from *Astyanax lacustris*, employing 0.1% sodium dodecyl sulfate (SDS) in conjunction with physical agitation. The efficacy of cellular removal was confirmed by DNA quantification, histological evaluation and DAPI staining, whereas the preservation of ECM integrity was validated through immunofluorescence, scanning electron microscopy (SEM), transmission electron microscopy (TEM), and proteomic profiling. SDS treatment effectively eliminated cellular components while preserving key ECM proteins, including Collagen I, Fibronectin, and Laminin α1. Notably, critical ultrastructural features—such as the basal lamina, seminiferous tubules, and the D-periodic banding pattern of collagen fibrils—were retained post-decellularization. Proteomic analyses revealed enrichment of proteins associated with ECM organization, cell adhesion, and collagen biosynthesis, while proteins involved in glycolysis and metabolic pathways were downregulated. Moreover, the decellularized matrix retained a comprehensive repertoire of matrisome components, including multiple collagen subtypes (Col1, Col2, Col4, Col5, Col6, and Col7), glycoproteins (Fibronectin, Laminin), proteoglycans (Heparan sulfate), ECM-affiliated proteins (Integrins), secreted factors (Collagen- and calcium-binding EGF), and ECM regulators (Glycosaminoglycans). Collectively, these findings demonstrate that our protocol effectively preserves the structural and functional hallmarks of the testicular ECM, underscoring its potential as a biologically relevant scaffold for future applications in fish reproductive biology. Further investigations are warranted to optimize hydrogel formulations and assess their capacity to support the *in vitro* proliferation and differentiation of spermatogonial stem cells (SSCs).

## 1 Introduction

The development of decellularized extracellular matrix (dECM) has profoundly advanced 3D cell culture by providing a biomimetic microenvironment that closely mimics *in vivo* conditions (Crapo et al., 2011; Brown and Badylak, 2014; Porzionato et al., 2018; Guruswamy Damodaran and Vermette, 2018; Giobbe et al., 2019; Bashiri et al., 2021; Cho et al., 2021; Talaei-Khozani and Yaghoubi, 2022; Heo et al., 2022; Horvath-Pereira et al., 2023; Fernández-Pérez and Ahearne, 2019). Generally, ECM is a combination of tissue-specific growth factors and secreted cellular elements such as fibrous proteins (collagen and elastin), adhesive glycoproteins (laminin and fibronectin), and proteoglycans important for mechanical resistance to compressive forces, strength, and attachment (Mouw et al., 2014; Häcker et al., 2005; Schaefer and Schaefer, 2010; Schwarzbauer and DeSimone, 2011; Kim et al., 2011; Padhi and Nain, 2020). This unique capacity of ECM to retain native biochemical and biophysical cues renders highly effective support for tissue-specific cell function, especially in a complex process such as spermatogenesis (Siu and Cheng, 2009; Cheng and Mruk, 2010; Li et al., 2021; Tosti and Ménézo, 2016; Bu et al., 2022).

As the ECM undergoes significant remodeling during the reproductive cycle, spermatogenesis relies on these ECM components to support testicular cells to organize and interact in an appropriate manner (Santana and Quagio-Grassiotto, 2014; O'Donnell et al., 2015; Li et al., 2002). Such dynamic remodeling of the ECM highlights its role not only as a structural support but also as a key regulator of cell adhesion, migration, proliferation, and differentiation (Lin et al., 2012; Gharenaz et al., 2020; Kiani et al., 2021). Given these advantages, recent efforts have been directed toward developing and characterizing natural decellularized testicular extracellular matrix (dtECM) scaffolds to advance *in vitro* spermatogenesis and support research on fertility preservation (Baert et al., 2015; Baert et al., 2017; Topraggaleh et al., 2019; Vermeulen et al., 2019; Salem et al., 2023).

Baert et al. (2015) made the initial significant advancements in this area by decellularizing human testicular tissue capable of supporting spermatogonial stem cell (SSC) testicular adhesion. Building on this, Baert et al. (2017) further recellularized these scaffolds with adult and pubertal testicular cells, generating functional testicular organoids (TOs) capable of supporting germ cell proliferation while the testis-specific cytoarchitecture was not restored. Based on these findings, Vermeulen et al. (2019) advanced this approach using dtECM hydrogel to generate testicular porcine organoids, which not only enabled the development of seminiferous tubule-like structures but also led to spermatogenesis progression to postmeiotic stages. Furthermore, other studies have reported that dtECM hydrogels could improve SSC differentiation and stimulate spermatid markers along with testosterone and inhibin secretion (Topraggaleh et al., 2019; Salem et al., 2023; Naeemi et al., 2021; Yang et al., 2020). Therefore, these complementary studies demonstrate the versatility of dtECM scaffolds to facilitate the reorganization of 3D cell aggregates, ultimately forming a compartmentalized testis-derived organoid that closely resembles the native microenvironment and supports spermatogenesis.

Although dECM-based scaffolds are already widely applied in mammalian organoid culture (Vermeulen et al., 2019; Salem et al., 2023; Naeemi et al., 2021; Yang et al., 2020; Cham et al., 2021; De Vriendt et al., 2023; Chen et al., 2024; Garreta et al., 2024; Bhattacharya et al., 2024), standardized protocols for developing fish-specific dECM models have not yet been established. For that, *Astyanax lacustris*, a Neotropical characin fish, was selected since it is gaining recognition as a model species for reproductive and developmental studies due to its numerous favorable biological and ecological traits (Adolfi et al., 2015; Siqueira-Silva et al., 2021; de Paiva Camargo et al., 2017; Martinez-Bengochea et al., 2020; Branco et al., 2021; Postingel et al., 2021; Yasui et al., 2022; Branco et al., 2025). Its small size, ease of adaptation to laboratory and aquaculture environments, early sexual maturity, and year-round breeding potential under controlled conditions make it highly suitable for experimental applications. These features enable advanced reproductive biotechnologies such as germ cell transplantation and the development of testicular scaffolds, which are critical for understanding and supporting germ cell development (Yasui et al., 2022). Therefore, the current study aimed to establish a protocol for developing a dtECM in *A. lacustris* that preserves the three-dimensional ECM structure and the tissue-specific components essential for *in vitro* 3D culture. By developing this dtECM protocol, we could greatly expand research applications in reproductive biology to improve *in vitro* spermatogenesis of economically important fish species, as well as in toxicology and disease modeling studies.

## 2 Materials and methods

### 2.1 Animals

Adult male yellowtail tetra (*A. lacustris*) (n = 250) were used as the experimental model in this study. For this purpose, the fish were kept in a 500 L tank with a recirculation system (28°C; pH 7.6; conductivity of 750 μS) under a 14 h:10 h (light, dark) photoperiod at the aquarium facility of the Department of Structural and Functional Biology of the Institute of Biosciences, UNESP-Botucatu. All experiments were conducted in accordance with the Guide for the Care and Use of Laboratory Animals (National Research Council) and were approved by the Ethics Committee on Animals Experiments of São Paulo State University (UNESP), protocol number 4577270922-CEUA.

### 2.2 Decellularization of testicular tissue fragments

Following *A. lacustris* euthanasia with benzocaine solution (0.1 g L^-1^), testicular tissues were excised, sectioned into ∼0.5 mm^3^ fragments as described by Miura et al. (1991) and Oliveira et al. (2021), and washed with 1X phosphate-buffered saline (PBS, pH 7.4). For decellularization, this study systematically evaluated the use of sodium dodecyl sulphate (SDS) (Khazaei et al., 2023; Gupta et al., 2018; Doretto et al., 2022) under agitation (2000 rpm) at room temperature and identified 0.1% SDS as the optimal concentration. Control samples were rinsed in 1X PBS under the same experimental conditions and for the same duration as the treated samples, but without the addition of SDS, ensuring a valid baseline for comparison. Following decellularization, all samples were subjected to eight sequential 30-min washes in 1X PBS, followed by overnight storage (12 h) in 1X PBS at 4°C to ensure thorough removal of cellular debris and residual SDS (Khazaei et al., 2023; Xu et al., 2014).

To further refine the decellularization protocol, the study investigated the influence of varying detergent exposure durations (2, 4, and 6 h), enabling the optimization of conditions that maximize cellular removal efficiency while preserving the integrity and functionality of the extracellular matrix. Posteriorly, the 2-h protocol was selected, and the preservation of ECM functionality was thoroughly validated using various methods. This included assessing tissue microarchitecture and ultrastructure, performing immunofluorescence staining for essential ECM proteins (such as collagen, fibronectin, and laminin), and conducting proteomic profiling to ensure comprehensive evaluation.

### 2.3 Evaluation of the decellularization procedure

#### 2.3.1 Histological analysis

To assess the efficiency of the decellularization process and the extracellular matrix (ECM) preservation, both control (PBS) and decellularized (0.1% SDS) testicular fragments were randomly selected for histological evaluation (n = 10 replicates per group). Control and decellularized samples were fixed in Karnovsky’s fixative (2% glutaraldehyde and 4% paraformaldehyde in Sorensen buffer, pH 7.4) for 24 h at room temperature. Following fixation, samples were dehydrated through a graded ethanol series, embedded in Technovit 7,100 historesin (Heraeus Kulzer, Wehrheim, Germany), and sectioned to a thickness of 5 μm. Hematoxylin and eosin (HE) staining was performed to confirm the absence of cellular components, and Masson’s trichrome staining was used to visualize collagen fibers. The stained slides were examined using a Leica DMI6000 microscope (Leica, Heidelberg, Germany). Based on the histological analysis, testicular fragments treated with 0.1% SDS and PBS (control) for 2 h were selected for further analysis through scanning electron microscopy, transmission electron microscopy, and proteomic analysis.

#### 2.3.2 DNA extraction and purity assessment

Genomic DNA was isolated from both control (1% PBS-treated) and decellularized testicular ECM (0.1% SDS-treated) samples (one testis lobe) using the DNeasy Blood & Tissue Kit (QIAGEN). Following tissue digestion with proteinase K (56°C, 3 h), DNA was purified via ethanol precipitation and DNeasy spin columns. Purified DNA was eluted and assessed for purity by spectrophotometry (Nanodrop Technologies Inc., Wilmington, United States; 260/280 nm absorbance ratio.

#### 2.3.3 Immunofluorescence analysis

Immunofluorescence was employed to evaluate the extracellular matrix (ECM) composition and assess the preservation of ECM components after decellularization. Key matrix proteins, including collagen type I, fibronectin, and laminin α1, were analyzed in control (1X PBS) and decellularized (0.1% SDS) testicular fragments (n = 10 replicates per condition). This comprehensive approach aimed to determine whether decellularization treatment effectively preserved the ECM by retaining critical components while ensuring efficient cellular removal.

Therefore, decellularized fragments (0.1% SDS, 2 h) and control samples (PBS-treated) were fixed in 4% paraformaldehyde in 1X PBS for 1 h at room temperature (RT). After fixation, samples were rinsed and stored in 1X PBS containing 0.05% (w/v) sodium azide (Sigma-Aldrich) at 4°C until further analysis. The samples were dehydrated, embedded in paraffin and sectioned at 5 µm thickness. Histological sections were first deparaffinized and rehydrated, followed by treatment with sodium borohydride (NaBH_4_; Sigma Aldrich, San Luis, MI, United States) for 3 min to reduce background fluorescence following previously established protocol (Doretto et al., 2022). Sections were then blocked with 3% bovine serum albumin (BSA) before being incubated overnight at 4°C with goat anti-collagen type I (1:200, Santa Cruz-8788), goat anti-laminin α1 (1:200, Santa Cruz-6017), and mouse anti-fibronectin (1:100, Santa Cruz-8422). Following incubation, samples were rinsed with 1X PBS and incubated with donkey anti-goat IgG H&L (Alexa Fluor^®^ 488) (1:400, Abcam-150129) for collagen type I and laminin α1, and goat anti-mouse IgG H&L (Alexa Fluor^®^ 568) (1:400, Abcam-175473) for fibronectin for 1 h at RT. After washing with 1X PBS, samples were incubated with 1 μg/mL DAPI for 10 min at room temperature to visualize nuclei. Imaging was carried out by using a Leica DM6000 BD (Leica Microsystems, Wetzlar, Germany).

### 2.4 Microarchitecture and ultrastructure characterization

To assess the microarchitecture and ultrastructure of control and decellularized testicular fragments, both scanning electron microscopy (SEM) and transmission electron microscopy (TEM) were utilized. For SEM analysis, decellularized fragments (0.1% SDS, 2 h) and control samples (PBS-treated) were initially fixed in 2.5% glutaraldehyde for 24 h at 4°C and subsequently washed with 0.1 M PBS. Samples were post-fixed with 1% osmium tetroxide (O5500, Sigma-Aldrich) in 0.1 M PBS (pH 7.3) for 2 h at RT, followed by dehydration through a graded ethanol series and drying with hexamethyldisilazane. The samples were then sputter-coated with gold using a Q150R-ES coater (Quorum Technologies, United Kingdom) and imaged with a VEGA3 scanning electron microscope, enabling detailed evaluation of surface topography and structural preservation. For TEM, both control (PBS-treated, n = 5) and decellularized (0.1% SDS, 2 h, n = 5) testicular fragments were fixed in 2% glutaraldehyde (Sigma, St. Louis, MO) in cacodylate buffer for 30 min at room temperature, post-fixed in 1% osmium tetroxide for 1 h, washed with PBS, and dehydrated through an acetone series before embedding in Araldite. Ultrathin sections (50–75 nm) were cut using a diamond knife on a Leica Ultracut UCT ultramicrotome and collected onto carbon/formvar-coated grids. Sections were post-stained with uranyl acetate and lead citrate to enhance contrast and imaged using a LEO–Zeiss 906 TEM operating at 80 kV. These complementary imaging techniques provided high-resolution insights into the preservation of ECM architecture and the impact of decellularization on tissue ultrastructure, ensuring a thorough evaluation of the testicular matrix integrity.

### 2.5 Proteomics

#### 2.5.1 Protein extraction, quantification and digestion

Proteins were extracted from control (PBS-treated) (n = 50, 4 replicates) and decellularized fragments (0.1% SDS, 2 h) (n = 50, 4 replicates) using an extraction buffer (8 M urea, 100 μL; 1 M Tris; 100 mM PHSF 1% protease inhibitor; 65 mM DTT) for 5 min. The resulting homogenate was subjected to ultrasonication in ultrapure water at 4°C for 5 min and centrifuged at 14,000 rpm at 4°C. This procedure was repeated three times. Supernatants were collected, and total protein content was quantified using the Bradford assay, following the manufacturer’s instructions (Quick Start™ Bradford Protein Assay Kit, Bio-Rad, Hercules, CA, United States). Proteins (400 µg) from each sample were diluted in 60 µL 50 mM ammonium bicarbonate and mixed with 25 µL 0.2% Rapigest. After incubation (37°C, 60 min), samples underwent reduction (10 mM DTT) and alkylation (45 mM IAA) in 50 mM ammonium bicarbonate. Trypsin (1:50) digestion followed (37°C, 16 h, pH 7.8). The reaction was quenched with 10 µL 5% trifluoroacetic acid. Samples were desalted using Sep-Pak Vac C18 cartridges (Waters, Milford, MA, United States), then concentrated using a SpeedVac™ (Thermo Scientific, Waltham, MA, United States) and stored at −20°C for subsequent analysis via liquid chromatography-tandem mass spectrometry (LC–MS/MS).

#### 2.5.2 Liquid chromatography-tandem mass spectrometry (LC–MS/MS) analysis

Peptide analysis was conducted via LC–MS/MS using data-independent acquisition (MSE) on a nanoUPLC-Synapt G2-Si HDMS system (Waters, Manchester, United Kingdom). Raw mass spectrometry data were processed with ProteinLynx Global Server (PLGS v3.00) software with a standardized workflow. Initially, mass calibration was applied using Glu1-Fibrinopeptide B (m/z 785.8426) as lock mass, followed by ion detection and chromatographic peak characterization via Apex3D algorithm. Peptide3D then deconvoluted multiply charged ions into monoisotopic masses and clustered them into Accurate Mass Retention Time (AMRT) features. Protein identification was achieved through ion accounting against a target database (trypsin digestion, 1 missed cleavage allowed) with mass tolerances of 10 ppm (precursor) and 20 ppm (fragment ions), applying a 4% false discovery rate (FDR) threshold via target-decoy validation. For label-free quantification, data were normalized by total ion current (TIC) alignment, with relative protein abundance calculated using the Hi-N algorithm (top 3 peptides/protein). Differential expression was determined using a modified t-test, considering proteins with p < 0.05 as downregulated and p > 0.95 as upregulated. (Fioretto et al., 2024). Only proteins detected in ≥3 of 4 biological replicates per group were included in the final analysis. Unique proteins appearing exclusively in one condition were annotated separately. Chromatographic alignment was optimized using Monte Carlo algorithms, and technical variability was assessed via coefficient of variation (CV) of peptide intensities across replicates.

Protein identification was performed by searching the MS/MS data against the *Astyanax mexicanus* database (https://www.uniprot.org/), with “uncharacterized” proteins further confirmed and classified using the zebrafish (*Danio rerio*) UniProt database (https://www.uniprot.org/). Differentially expressed proteins identified through shotgun proteomics underwent pathway enrichment analysis via the Kyoto Encyclopedia of Genes and Genomes (KEGG) and gene ontology (GO) for molecular function, biological process, and cellular component categories, using the ShinyGO v0.80 bioinformatics tool (http://bioinformatics.sdstate.edu/go/). The analysis used a hypergeometric test followed by Fisher’s exact test, with FDR correction applied using the Benjamini-Hochberg method. Protein-protein interaction (PPI) networks were generated using STRING database (https://string-db.org) v11.0 with a medium confidence threshold of 0.4.

Matrisome annotations were determined using Matrisome AnalyzeR (http://matrisomedb.pepchem.org/; *in silico* zebrafish matrisome) (Nauroy et al., 2018). Identified proteins were categorized into six divisions: collagens, ECM glycoproteins, proteoglycans, ECM-affiliated proteins, ECM regulators, and secreted factors. The matrisome content and composition in native and decellularized testicular extracellular matrix (dtECM) were then compared based on relative protein abundance, and further enrichment analysis of upregulated matrisome proteins was conducted.

## 3 Results

### 3.1 Histological analysis and DNA content of testicular scaffolds

**FIGURE 1:**
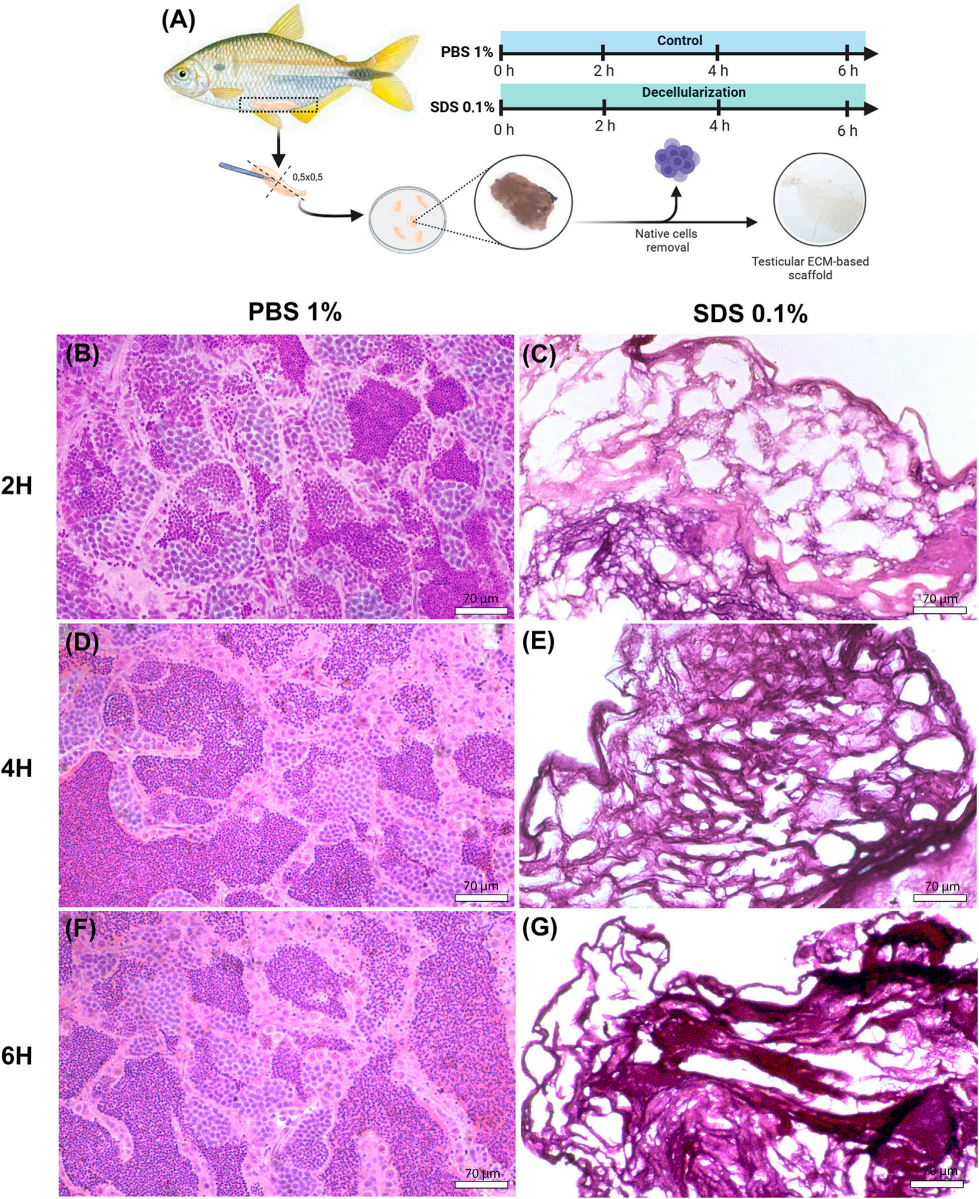
Histological analysis of native and decellularized testicular ECM (dtECM) **(A)** Schematic representation of the testicular decellularization process using 0.1% SDS for 2, 4, or 6 h under agitation. Native testis was treated with 1% PBS as a control. Following each treatment, translucent fragments were selected for histological analysis **(C,E,G)**. Hematoxylin and eosin (H&E) staining of dtECM shows effective cell removal while preserving the structural integrity of the seminiferous tubules. In contrast, the native testis shows seminiferous tubules populated with germ cells at different stages of development **(B,D,F)**. Scale bar = 70 µm.

In this study, the testicular tissue of yellowtail *tetra* (*A. lacustris*) was decellularized using a combination of physical and chemical methods. Various exposure times were tested to optimize the removal of cellular debris while preserving the extracellular matrix integrity (Figure 1A). Histological analysis was performed to evaluate the efficacy of decellularization and identify the most effective protocol for preserving tissue structure (Figures 1B–G). The results indicated that testicular fragments treated with 0.1% SDS for 2, 4, or 6 h were fully decellularized, with seminiferous tubules visibly devoid of cellular content in all decellularized scaffolds (Figures 1C,E,G), in contrast to the control group, which remained intact cellular components (Figures 1B,D,F). However, the structural assessment revealed that longer exposure times, specifically 4 and 6 h, were more aggressive and induced slight alterations in tissue architecture when compared to the 2-h treatment. This effect was particularly pronounced in the 6-h treatment, where the outlines of the seminiferous tubules appeared irregular compared to the other conditions and the control (Figures 1F,G). These findings suggest that while all tested durations achieved effective decellularization, the 2-h exposure to 0.1% SDS provides a better balance between cellular removal and preservation of tissue integrity. Based on these histological findings, testicular fragments treated with 0.1% SDS for 2 h and its respective control (PBS-treated) were selected for further analysis.

To quantitatively complement our DAPI staining results, we measured residual DNA content in decellularized testicular ECM (dtECM) and control testis. Our analysis revealed a 99.99% reduction in dtECM DNA (0.0183 ± 0.00559) compared to native tissue (6,171 ± 227 ng/mg; p < 0.0001), confirming effective nuclear material removal (Supplementary Figure S1). The minimal residual cell debris and DNA indicated that the testicular tissue was successfully decellularized.

### 3.2 Integrity of ECM key proteins: collagen type I, laminin α1, and fibronectin

**FIGURE 2:**
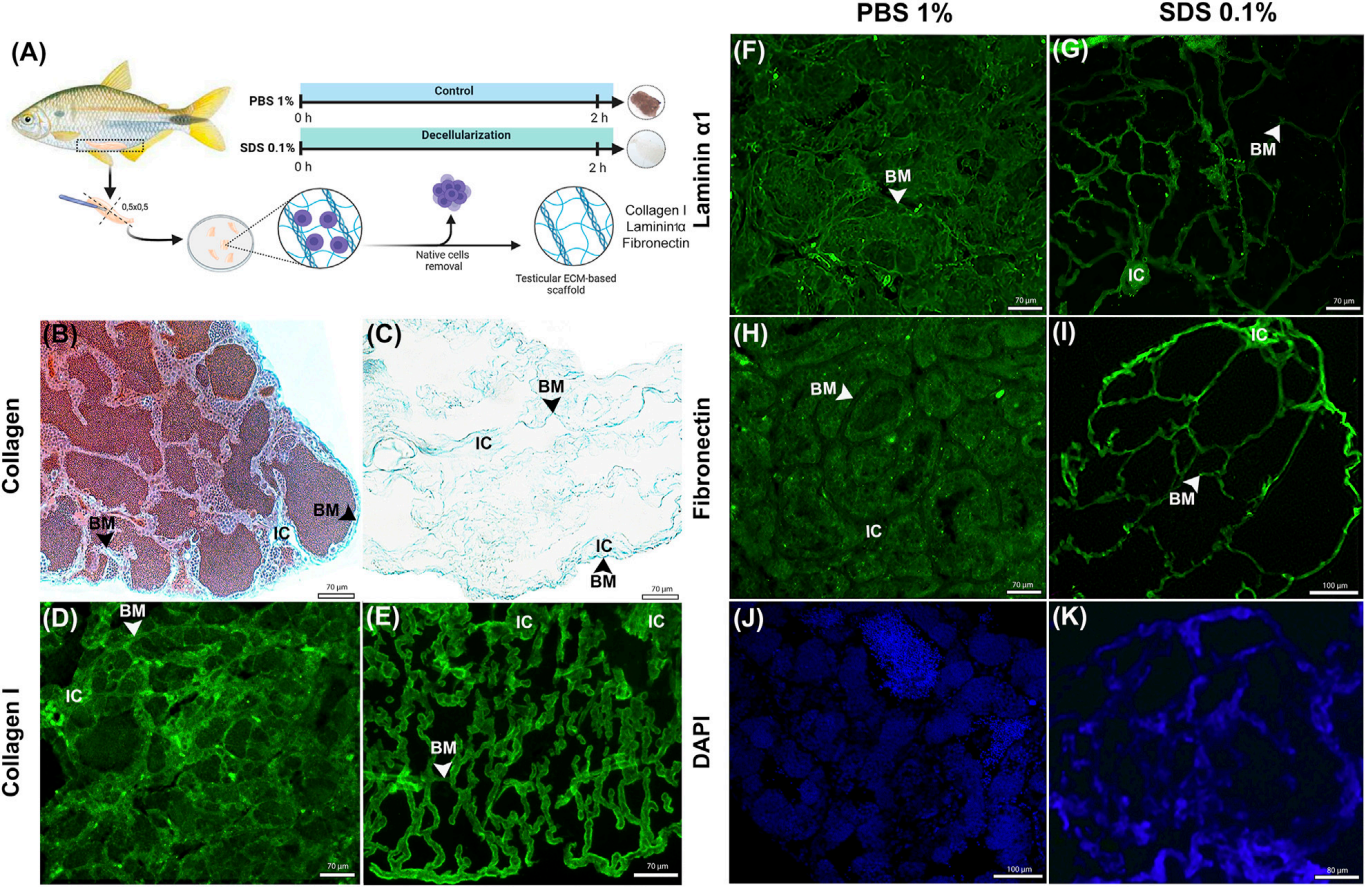
Histological and Immunofluorescence Analysis of ECM Proteins in Native and Decellularized Testis ECM (dtECM) **(A)** Schematic representation of the decellularization process using 0.1% SDS for 2 h. Native testis treated with 1% PBS served as control. Following decellularization, translucent tissue fragments were selected for ECM protein retention using histological and immunofluorescence methods. Masson trichrome staining **(C)** confirmed the presence of Collagens and immunofluorescence analysis confirmed the presence of Collagen type I **(E)**, Laminin α1 **(G)** and Fibronectin **(I)** in the dtECM (, showing retention patterns comparable to those in native testis **(B,D,F,H)**. DAPI staining confirmed the absence of nuclei in dtECM samples **(K)**, while native testis exhibited seminiferous tubules populated with different germ cells **(J)**. Scale bar = 70 µm. BM: basal membrane; IC: Interstitial compartment.

To evaluate the integrity of key ECM proteins following decellularization, Masson’s Trichrome (MT) staining for collagen and immunofluorescence detection for collagen type I, laminin α1, and fibronectin were conducted (Figures 2A–I). MT staining confirmed the complete removal of cellular content while demonstrating that collagens, a primary ECM component critical for structural integrity (Frantz et al., 2010; Fratzl and Fratzl, 2008) remained preserved following decellularization (Figure 2C). Furthermore, the histoarchitecture of seminiferous tubules was well-maintained after SDS treatment, showing similarity to the control group (Figure 2B). Immunostaining results highlight the retention of collagen type I in decellularized testicular fragments (Figure 2E). Collagen type I was found in the interstitial compartment and surrounding the seminiferous tubules of *A. lacustris* testes in both control and decellularized fragments (Figures 2D,E).

Additionally, laminin α1 and fibronectin, essential ECM components involved in cell adhesion and structural support (Aumailley, 2013; Zollinger and Smith, 2017) were observed to be preserved in decellularized testicular fragments as compared to control (Figures 2F,I). In *A. lacustris* testes, laminin α1 was localized along the basal membrane of the seminiferous tubules, delineating the entire tubular contour in both control and decellularized testicular fragments (Figures 2F,G). In contrast, fibronectin was also identified in the basal membrane but displayed a more dispersed distribution, extending into certain regions of the interstitial compartment (Figures 2H,I).

DAPI staining further confirmed the effectiveness of the decellularization protocol in removing germ cells within the seminiferous tubules. Compared to the control group, which showed abundant nuclear content (Figure 2J), SDS treatment resulted in complete germ cell removal (Figure 2K). Although DAPI stained the decellularized extracellular matrix, this could be attributed to nonspecific binding or residual nucleic acids adhering to the matrix. These findings demonstrate that the 0.1% SDS decellularization protocol effectively removes cellular components while preserving the structural integrity of the ECM. Key structural and adhesive proteins, including collagen type I, laminin α1, and fibronectin, remained intact, ensuring the functionality and applicability of decellularized testicular scaffolds for downstream applications.

### 3.3 Microarchitecture and ultrastructure integrity of ECM

**FIGURE 3:**
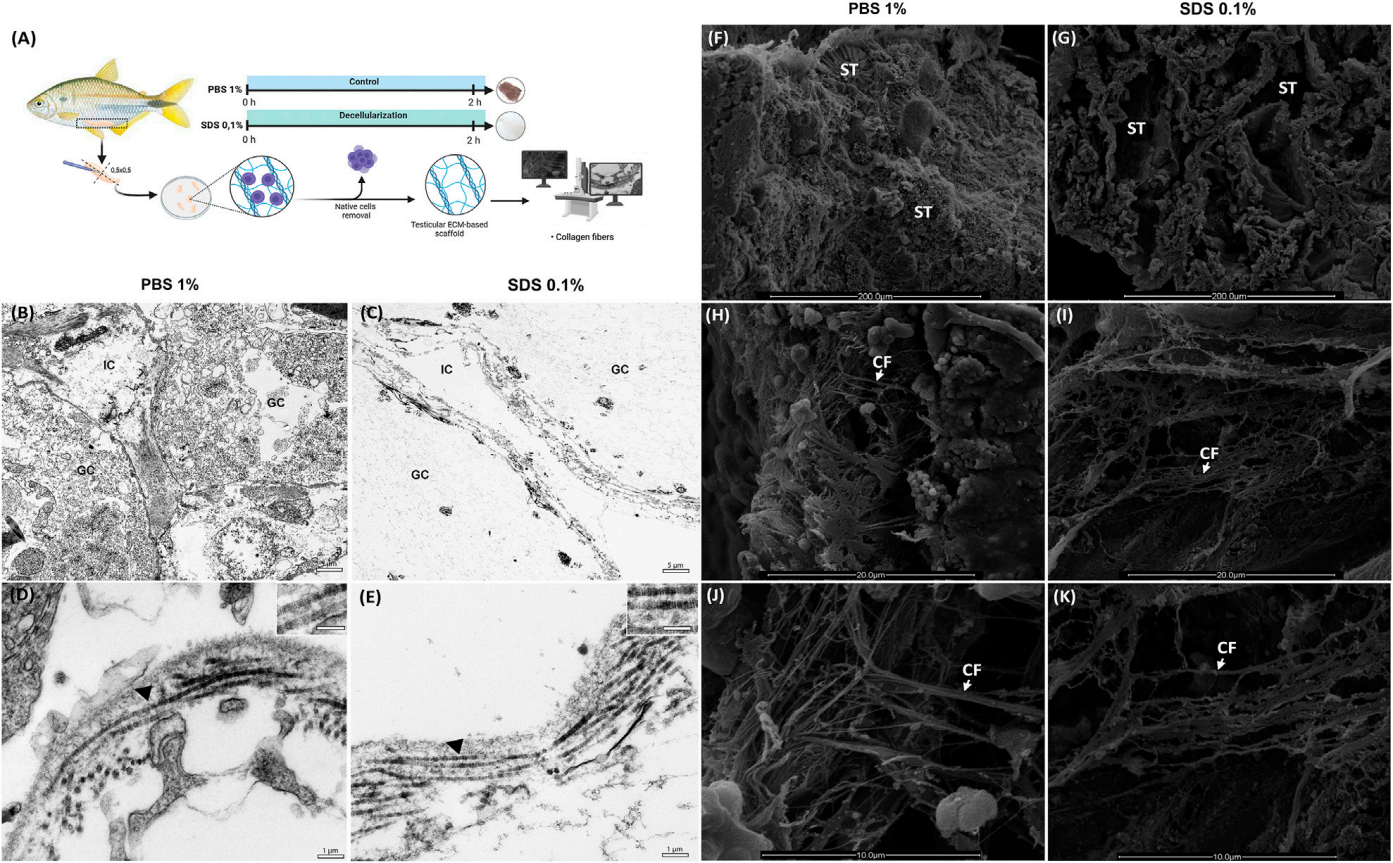
Ultrastructural Analysis of Decellularized Testicular Extracellular Matrix (dtECM) and Native Testis **(A)** Schematic representation of the decellularization process using 0.1% SDS for 2 h. Native testis treated with 1% PBS served as control. Following decellularization, translucent tissue fragments were selected for Transmission electron microscopy (TEM) and Scanning electron microscopy (SEM) methods. TEM highlights the structural integrity of interstitial compartments and seminiferous tubules in both dtECM and native testis **(B,C)**. Notably, collagen fibrils in dtECM retained their characteristic D-periodic banding **(E)**, displaying high structural similarity to native testis **(D)**. IC: interstitial compartment; GC: germinal compartment. Black arrowheads indicate D-periodic bands of collagen fibrils. Scale bars: **(A,B)** 5 μm; **(C,D)** 1 µm. SEM of native testis (1% PBS) reveals seminiferous tubules densely populated with cells **(F)**, whereas at higher magnification, SEM of dtECM shows seminiferous tubules completely devoid of cellular content **(G)**. The orientation and structural integrity of collagen fibrils in dtECM **(I,K)** were preserved, closely resembling the collagen organization observed in native testis **(H,J)**. ST: seminiferous tubules; CF: collagen fibrils. Scale bars: **(A,B)** 200 μm; **(C,D)** 20 μm; **(E,F)** 10 µm.

The three-dimensional (3D) microarchitecture and ultrastructure of testicular scaffolds were qualitatively assessed (Figure 3A) using TEM (Figures 3B–E) and SEM (Figures 3F–K), respectively.

TEM analysis provided complementary insights into the ultrastructure of the testicular tissue. In the control testicular fragments (PBS-treated), the germinative compartment exhibited various developing germ cells, while the interstitial compartment contained interstitial cells (Figure 3B). SDS treatment effectively eliminated all cellular components from both compartments, leaving behind an extracellular matrix (ECM) scaffold devoid of cells (Figure 3C). The collagen fibrils in the decellularized samples remained comparable to the control, showing orthogonal orientation and preserved D-periodic bands (Figures 3D,E), indicating that SDS treatment did not compromise the ultrastructure of the collagen fibrils.

Moreover, SEM analysis revealed that the overall architecture of the ECM was preserved following decellularization (Figure 3G). Both the native control (PBS-treated) and decellularized testicular fragments (treated with 0.1% SDS for 2 h) displayed well-defined seminiferous tubules (Figures 3F,G). At lower magnifications, germ cell cysts were observed within the seminiferous tubules of control fragments but were effectively removed in SDS-treated samples, leaving the ECM intact (Figures 3H,I). Higher magnification images highlighted the collagen fiber network in the testicular microarchitecture of the control fragments, particularly in the testicular capsule and interstitial compartments (Figures 3J,K). Notably, treatment with 0.1% SDS for 2 h preserved the collagen network’s microarchitecture, maintaining the structural integrity of the ECM and its fibrils (Figures 3J,K).

### 3.4 Global proteomics identification and enrichment analysis

**FIGURE 4:**
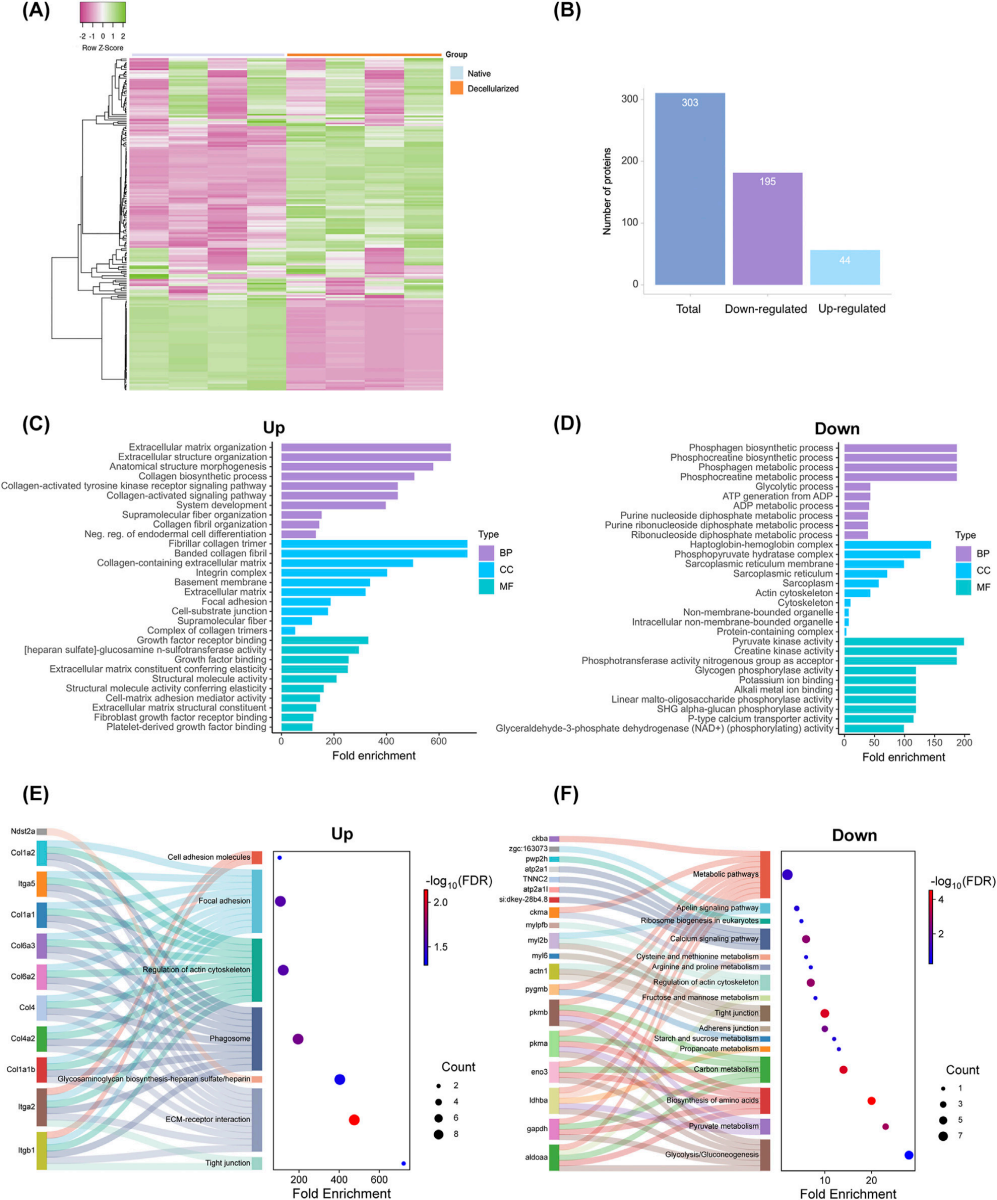
Proteomic Analysis of Native and Decellularized Testicular Extracellular Matrix (dtECM) **(A,B)** Total protein identification and classification of proteins exhibiting increased (upregulated) or decreased (downregulated) abundance in native and dtECM samples. Gene Ontology (GO) enrichment analysis highlights the top 10 significantly enriched terms for proteins with elevated **(C)** and reduced **(D)** abundance, categorized into Biological Process, Cellular Component, and Molecular Function. Bar lengths represent fold enrichment, with significance indicated by an FDR-adjusted p-value ≤0.05. **(E,F)** Functional and pathway enrichment analyses of differentially abundant proteins (DAPs) between native and decellularized testis ECM. Sankey plots illustrate specific pathways enriched by proteins with increased **(E)** and decreased **(F)** abundance. The gene names associated with each pathway are connected by lines, with dot sizes representing the number of genes and color gradients indicating significance, ranging from blue (low significance) to red (high significance). False discovery rates (FDR-adjusted p-value ≤0.05) were calculated using the Benjamini-Hochberg method to ensure statistical robustness.

**FIGURE 5:**
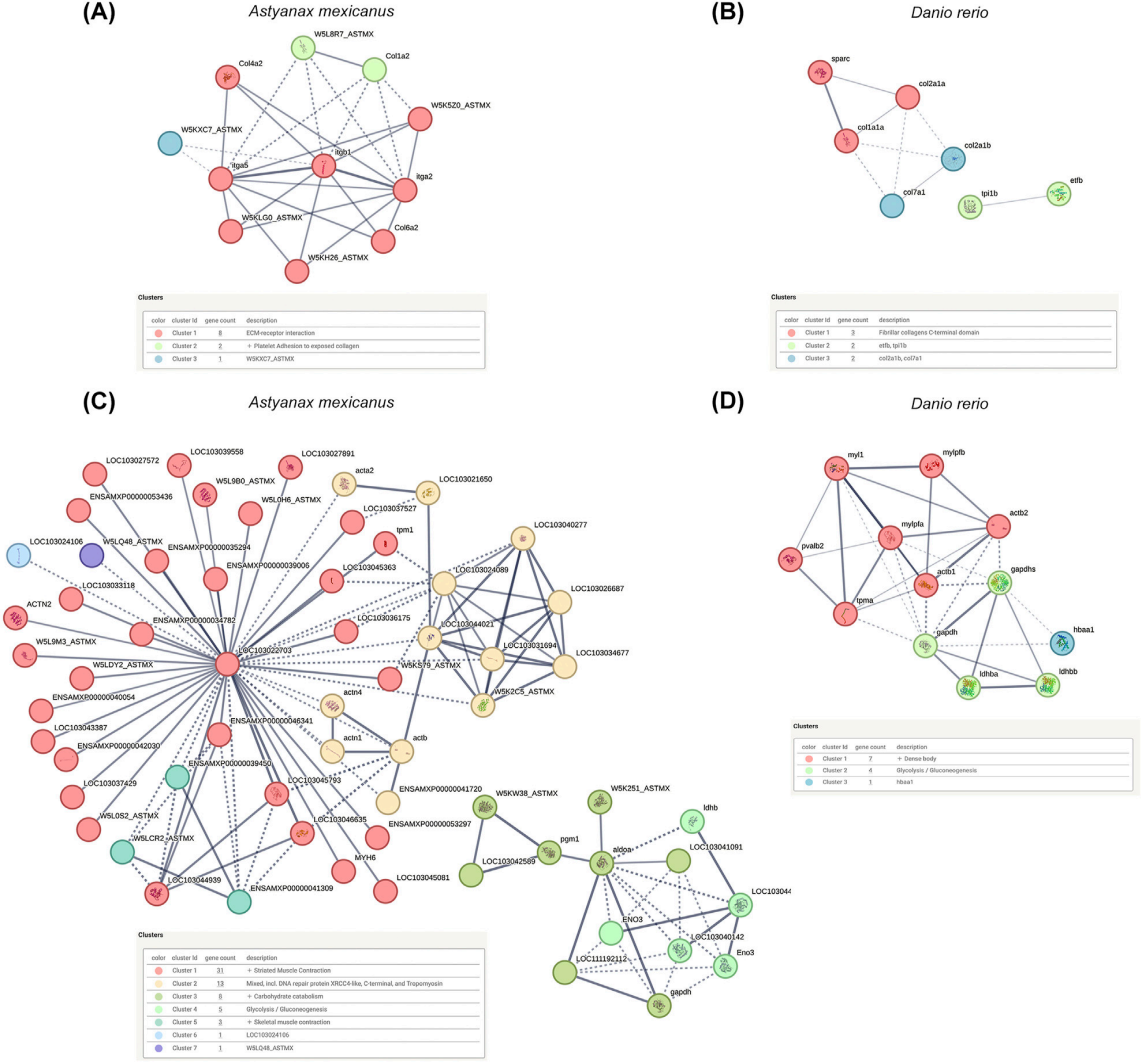
Protein-Protein Interaction (PPI) Networks of Differentially Abundant Proteins (DA Ps) **(A,B)** PPI networks constructed from differentially abundant proteins between decellularized testicular ECM (dtECM) and native testis, using their respective orthologs in *Astyanax mexicanus* and *Danio rerio*, reveal key interactions and clustering of proteins associated with extracellular matrix (ECM) structural integrity and adhesion-related pathways. **(C,D)** Following the same approach, PPI networks of depleted proteins—those with reduced abundance in dtECM—highlight disrupted interactions primarily affecting cellular processes such as metabolism and cytoskeletal organization. Nodes represent individual proteins, while edges (lines) denote connections based on a medium-confidence interaction score (score = 0.4). Functional clusters are color-coded to reflect distinct biological processes, providing insights into the structural and regulatory modifications induced by decellularization.

**FIGURE 6:**
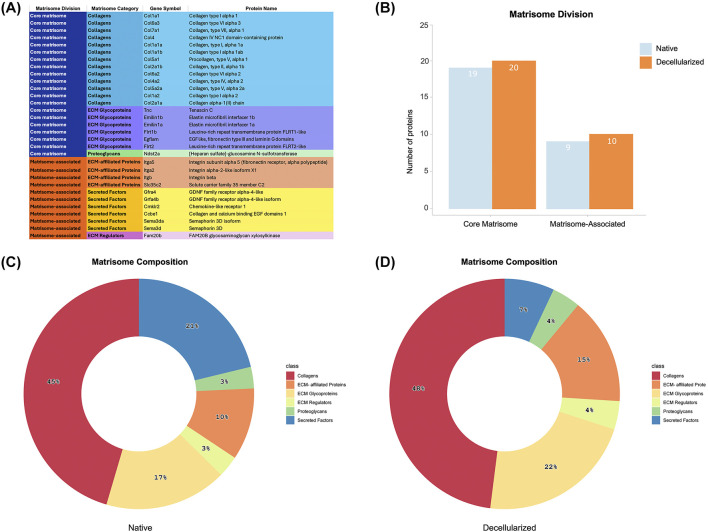
Matrisome Signature of Native and Decellularized Testicular ECM (dtECM) **(A)** The table categorizes matrisome proteins into core matrisome (Collagens, ECM Glycoproteins, and Proteoglycans) and matrisome-associated proteins (ECM-Affiliated Proteins, Secreted Factors, and ECM Regulators), along with their corresponding gene symbols. This classification provides a comprehensive framework for understanding the functional contributions of specific ECM components and how their retention or depletion is influenced by the decellularization process. **(B)** The total number of identified proteins within each matrisome category, distinguishing between core matrisome and matrisome-associated proteins. **(C,D)** Pie charts illustrate the proportional distribution of proteins across different matrisome subcategories in native testis ECM **(C)** and dtECM **(D)**, emphasizing compositional shifts following decellularization. These changes provide insights into the preservation of essential ECM components and the structural modifications induced by the removal of cellular elements.

**FIGURE 7:**
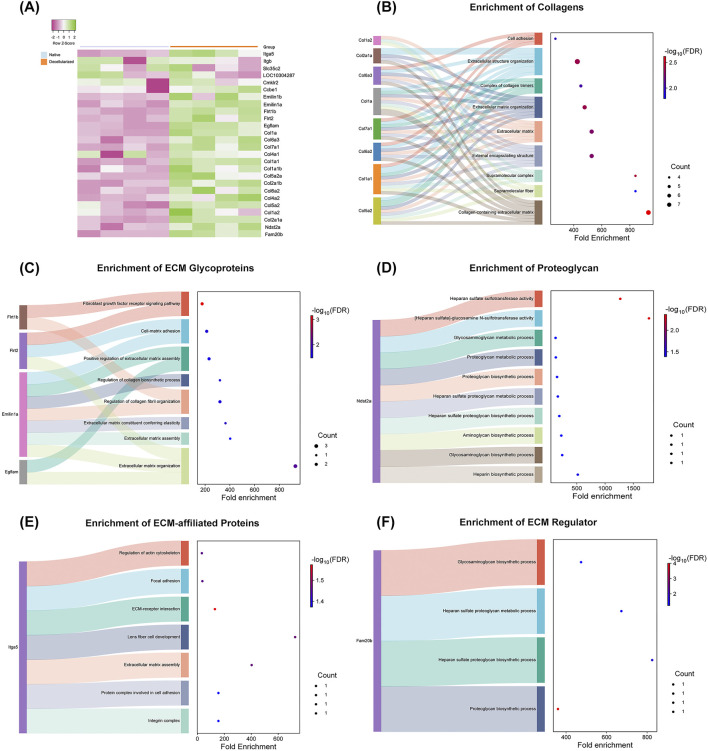
Functional Enrichment of Matrisome Proteins in Native and Decellularized Testicular ECM (dtECM) **(A)** Heatmap depicting the relative abundance of matrisome proteins in native and dtECM samples. Green indicates higher abundance in native testis, purple represents higher abundance in dtECM, and neutral tones (0) denote equivalent levels between the two groups. **(B–F)** Functional enrichment analysis of differentially abundant matrisome proteins (DEPs) between native and dtECM. The Sankey plot illustrates the enrichment of DEPs in pathways associated with the core matrisome **(B–D)** and matrisome-associated proteins **(E–F)**. Only proteins exhibiting a log_2_ fold change >1.5 in native testis are displayed, emphasizing those significantly altered in dtECM. Dot sizes correspond to the number of genes involved in each pathway, while the color gradient (blue to red) represents enrichment significance, with red indicating the highest statistical significance (FDR-adjusted p-value ≤0.05).

Proteomics assay was carried out to identify and evaluate differences in protein composition between native and decellularized testicular extracellular matrix (dtECM) scaffolds (Figures 4–7). As illustrated in Figures 4A,B, a total of 303 proteins were identified, including 239 differentially abundant proteins between native and dtECM scaffolds. Of these proteins, 44 were upregulated (i.e., enriched) while 195 were depleted in the dtECM compared to native tissue (Figures 4A,B). To further understand the functional roles of these differentially abundant proteins between native tissue and dtECM scaffolds, Gene Ontology (GO) analysis was conducted to assess enriched biological processes, molecular functions, and cellular components (Figures 4C,D). Functional enrichment of differentially abundant proteins highlighted distinct GO terms associated with up and downregulated proteins. Notably, the top 10 enriched biological process in the enriched proteins are primarily related to ECM, including ECM organization, collagen biosynthetic process, collagen-activated tyrosine kinase receptor, the supramolecular fiber complex and collagen fibril organization (Figure 4C). In terms of cellular components, the upregulated proteins were enriched in terms related to fibrillar and banded collagen trimer, collagen-containing ECM, integrin and collagen complexes, basement membrane and proteins involved in focal adhesion and cell-substrate junction (Figure 4C). For molecular function category, the enriched proteins were associated with the regulation of fibroblast and growth factor receptor binding, extracellular matrix constituent and structural molecule activity conferring elasticity and heparan sulfate and cell-matrix adhesion mediator activity (Figure 4C).

Further enrichment analysis using the KEGG pathway highlighted ECM-receptor interactions, glycosaminoglycan biosynthesis, cell adhesion molecules, regulation of actin cytoskeleton and focal adhesion as the most enriched pathways in upregulated proteins (Figure 4E; Supplementary Figures S2-S4). Remarkably, the enriched ECM components included a variety of collagen subtypes, proteoglycans (e.g., heparan sulfate) and glycoproteins (e.g., laminin domain, fibronectin, tenascin, emilin), as well as ECM-affiliated proteins such as integrins (Figure 4E). These results suggest that the decellularized scaffolds retain essential ECM biological components that may have an important role for testis function and spermatogenesis.

Among the proteins markedly depleted in the decellularized scaffolds, the most enriched biological processes were those related to phosphagen and phosphocreatine biosynthesis and metabolism, glycolysis, ATP generation from ADP, and the metabolic processing of purine nucleosides, purine ribonucleosides, and ribonucleoside diphosphates (Figure 4D; Supplementary Figures S5,S6). In terms of cellular localization, these reduced proteins were predominantly associated with the haptoglobin-hemoglobin complex, phosphopyruvate hydrates, the sarcoplasmic reticulum, and pyruvate kinase (Figure 4D). At the molecular function level, they were linked to creatine kinase activity, phosphotransferase activity, glycogen binding, and potassium ion binding (Figure 4D). Furthermore, KEGG pathway enrichment analysis showed their involvement in critical cellular pathways, including glycolysis/gluconeogenesis, amino acid biosynthesis, and general metabolic processes (Figure 4F).

### 3.5 Protein-Protein Interaction (PPI) network

Next, we conducted a Protein-Protein Interaction (PPI) analysis for the DAPs in the decellularized testicular scaffold compared to native testis, utilizing their respective orthologs in *A. mexicanus* and *D. rerio* (Figures 5A–D). For the enriched proteins in the decellularized testicular scaffolds, the PPI network identified protein clusters prominently involved in pathways related to cell adhesion, ECM-receptor interactions and ECM-associated proteins, fibrillar collagen formation, including collagens and integrins (Figures 5A,B). In contrast, the PPI analysis of the depleted proteins in the decellularized testicular scaffold identified protein clusters predominantly associated with metabolic processes and cellular structural components, reflecting the loss of cellular constituents following decellularization (Figures 5C,D). Major clusters were associated with elements related to energy metabolism and protein complexes such as glycolysis, gluconeogenesis, and muscle contraction processes, indicating that metabolic pathways and cytoskeletal components were significantly diminished in dtECM scaffolds. Overall, these PPI clustering results support the functional enrichments observed in the GO analysis, validating the preservation of essential ECM structures and cell adhesion functions within the dtECM while showing a marked reduction in cellular and metabolic activity in the scaffold.

### 3.6 Matrisome characterization

To gain deeper insight into the ECM proteome of decellularized testicular scaffolds, we employed the MatrisomeR (2.0) zebrafish database for protein identification (Figures 6A–D). This classification framework categorizes ECM components into the “core matrisome,” which includes collagens, proteoglycans, and glycoproteins, or in the “matrisome-associated” proteins, comprising secreted factors, ECM regulators, and ECM-affiliated proteins. By applying this approach, we delineated the specific matrisome signature enriched within the dtECM scaffolds. Utilizing this ECM-specific classification, we identified 32 distinct matrisome proteins across all decellularized scaffolds (Figure 6A). Among these, 20 proteins belonged to the core matrisome, while 10 were classified as matrisome-associated (Figure 6B). In comparison, native testis tissue contained 19 core matrisome proteins and 9 matrisome-associated proteins (Figure 6B).

Within the dtECM core matrisome, collagens were the most abundant, comprising 13 different types (48%), followed by glycoproteins (6 proteins, 22%) and proteoglycans (1 protein, 4%) (Figures 6A,D; Supplementary Figure S7A–C). The matrisome-associated category included 4 ECM-affiliated proteins (15%), 2 secreted factors (7%), and 1 ECM regulator (4%) (Figures 6A,D; Supplementary Figure S7A–C). These proportions closely mirrored those found in native testis tissue, which contained 13 collagens (45%), 5 glycoproteins (17%), and 1 proteoglycan (4%) in the core matrisome, alongside 6 secreted factors (21%), 3 ECM-affiliated proteins (10%), and 1 ECM regulator (4%) in the matrisome-associated category (Figure 6C; Supplementary Figure S7).

Comparing glycoproteins, ECM regulators, and ECM-affiliated proteins between dtECM and native testis tissue revealed that tenascin and one type of integrin (itga2) were uniquely present in the dtECM. Additionally, four secreted factors involved in immune cell responses (e.g., Semad3 and Semad3a) and spermatogonial proliferation (e.g., Gfra4 and Gfra4b) were exclusively detected in native testis tissue (Supplementary Figure S7).

To further dissect ECM composition, hierarchical clustering analysis of normalized relative abundance data was performed for all identified matrisome proteins (Figure 7A). Several collagen subtypes, including Col1a, Col1a1a, Col1a1b, Col2a1a, Col2a1b, Col4a1, Col4a2, Col5a2, Col6a2, Col6a3, and Col7a1, exhibited higher expression in dtECM, alongside glycoproteins such as elastin microfibrils (Emilin1a and Emilin1b), EGF-like/fibronectin, and laminin domain-containing proteins (Egflam, Flrt1b and Flrt2). Additionally, the ECM regulator glycosaminoglycan xylosylkinase (Fam20b), the proteoglycan heparan sulfate (Ndst2a), and integrins (Itga5) were also enriched in dtECM. Although laminin 1a was identified in decellularized testis tissue, it was excluded from further analysis due to a low confidence score.

To elucidate the functional roles of differentially abundant matrisome proteins in dtECM, enrichment analysis was conducted (Figures 7B–F). Among the core matrisome proteins of dtECM, Collagens including Col1, Col2, Col4, Col6, and Col7 were significantly enriched in pathways related to the collagen-containing ECM, supramolecular structure organization, and extracellular matrix remodeling (Figure 7B). Additionally, ECM glycoproteins such as fibronectin (Flrt1b and Flrt2), emilin (Emilin1a), EGF-like/fibronectin type III and laminin G domain-containing proteins (Egflam) were most enriched in fibroblast growth factor receptor signaling and ECM organization pathways (Figure 7C). Heparan sulfate (Ndst2a) (Figure 7D) exhibited significant enrichment in pathways associated with heparan sulfate sulfotransferase activity and heparan sulfate-glucosamine metabolism.

Within the matrisome-associated category (Figures 7E,F), integrins (Itga5) were prominently enriched in pathways related to ECM–receptor interactions, focal adhesion, and actin cytoskeleton regulation. Furthermore, the ECM regulator glycosaminoglycan (Fam20b) was enriched in biological processes related to proteoglycan metabolism, heparan sulfate biosynthesis, and glycosaminoglycan processing (Figure 7F).

## 4 Discussion

Decellularization has emerged as an effective approach for developing natural biological scaffolds that preserve ECM integrity and replicate the microenvironment necessary for germ cell attachment, development, and tissue regeneration (Porzionato et al., 2018; Guruswamy Damodaran and Vermette, 2018; Giobbe et al., 2019; Bashiri et al., 2021; Cho et al., 2021; Talaei-Khozani and Yaghoubi, 2022; Horvath-Pereira et al., 2023; Fernández-Pérez and Ahearne, 2019). The bioactive components of ECM scaffolds, including ECM proteins, growth factors, and adhesion proteins, play crucial roles in regulating germ cell proliferation and differentiation, thereby providing structural support to spermatogenesis (Cheng and Mruk, 2010; Li et al., 2021; Tosti and Ménézo, 2016; Bu et al., 2022; Naeemi et al., 2023; Bhaskar et al., 2021). Therefore, maintaining the structure and components of the ECM in dtECM-derived scaffolds is crucial for successful recellularization and tissue recapitulation.

Although dECM-based scaffolds are widely used in mammalian organoid culture systems across several tissue types (Cham et al., 2021; De Vriendt et al., 2023; Chen et al., 2024; Garreta et al., 2024; Bhattacharya et al., 2024) there are currently no standardized protocols for developing fish-specific dtECM models. Therefore, the current study developed a protocol to produce decellularized testicular extracellular matrix (dtECM) by applying a combined method using the ionic detergent SDS and physical agitation treatment. This approach aimed to balance effective cellular removal with minimal disruption of ECM integrity, thereby creating a scaffold that could support subsequent germ cell homing, attachment, and development.

In this study, the use of SDS effectively removed cellular debris from testis-derived dECM, as confirmed by DNA content, histological (HE and Masson’s trichrome) and DAPI staining analysis. This result meets established benchmarks for effective decellularization as previously demonstrated (Baert et al., 2015; Baert et al., 2017). Furthermore, the preservation of key ECM proteins (collagen type I, laminin α1, fibronectin) and the intact ultrastructure and microarchitecture of the testis, including the basal lamina, seminiferous tubules, and collagen fibril D-periodic banding were confirmed. These findings, consistent with our proteomic results, and align with prior mammalian studies where SDS-based protocols effectively preserved testicular ECM 3D architecture while eliminating cellular components (Gharenaz et al., 2020; Yang et al., 2020; Batista et al., 2022).

Proteomic profiles of decellularized scaffolds have been extensively characterized across various mammalian tissues, including small intestine and stomach (Giobbe et al., 2019), bone (Monteiro-Lobato et al., 2022), cornea (Hashimoto et al., 2024), vocal mucosa (Welham et al., 2013; Biehl et al., 2023), lung (Biehl et al., 2023; Li et al., 2016), pancreas (Hu et al., 2021), liver (Willemse et al., 2022; Diedrich et al., 2024), kidney (Diedrich et al., 2024; Zhang et al., 2021; Sobreiro-Almeida et al., 2021), heart (Biehl et al., 2023; Wang et al., 2021), skin (Biehl et al., 2023), and brain (Cho et al., 2021). However, research on dECM, particularly in testicular tissues, remains limited. Thus, to further characterize the dtECM, we conducted proteomic and functional enrichment analyses. Our results showed that ECM proteins crucial for tissue reconstruction were well preserved, whereas cytoplasmic and nuclear proteins significantly decreased in abundance. This result was confirmed by both enrichment and PPI network analysis, in which enriched proteins in the dtECM were mainly associated with ECM complexes, such as supramolecular fiber complexes, fibrillar collagen trimers, and collagen-containing ECMs. In contrast, depleted proteins were enriched in key cellular processes, including glycolysis/gluconeogenesis, amino acid biosynthesis, metabolic and troponin complexes.

To the best of our knowledge, only one study has analyzed decellularized human testis scaffolds through proteomic assays (Baert et al., 2015). Baert et al. (2015) identified ECM components such as collagens (I, IV, VI, XXI), glycoproteins (fibronectin, fibrillin, emilin, laminin), integrins, and secreted factors (e.g., estradiol-β-dehydrogenase, Wnt2b, EGF). In contrast, our study uncovered additional collagen subtypes (Col2, Col3, Col5, Col7) along with other ECM components such as the glycoprotein tenascin (Tnc), the glycosaminoglycan xylosyl kinase (Fam20b), and proteoglycan heparan sulfate (Ndst2a), which were not reported in human dtECM. While collagens I, III, IV, and V exhibit largely conserved roles in structural support and basement membrane integrity across mammals (Gatseva et al., 2019; Fidler et al., 2017; Ricard-Blum, 2011; Bosman and Stamenkovic, 2003) and fish (Panggabean et al., 2023), the presence of Collagen VII and tenascin in *A. lacustris* dECM, absent in human testes, suggests potential specialized functions, such as maintaining testicular tissue integrity under aquatic mechanical stresses and regulating germ cell development (Chung and Uitto, 2010; Anstrom and Tucker, 1996).

Further, we characterized the matrisome of both dtECM and native testis tissue (Nauroy et al., 2018), identifying fibrous proteins, including collagens (Col1, Col2, Col4, Col5, Col6, Col7) and elastin (Emilin1a, Emilin1b), which provide structural integrity (Siu and Cheng, 2009; Hohenester and Engel, 2002; Hynes, 2009; Theocharis et al., 2016). Adhesive glycoproteins such as tenascin (Tnc), fibronectin (Fltr1b, Flrt2), and laminin domains (Egflam) were also enriched, supporting basement membrane stability (Siu and Cheng, 2009; Bu et al., 2022; Kurek et al., 2021). Although laminin α1 showed low proteomic confidence, its presence was confirmed by immunofluorescence, and even small laminin fragments can retain integrin-binding functionality (Arimori et al., 2021). We also detected ECM regulators like Fam20b and Ndst2a, which modulate ECM biochemistry and hydrogel polymerization (Saldin et al., 2017; Beachley et al., 2018; Sasikumar et al., 2019). Additionally, dtECM preserved glycosaminoglycans and integrins (Itgb, Itga5), consistent with enriched ECM–receptor interactions and focal adhesion signaling pathways (Nauroy et al., 2018; Arimori et al., 2021; Taipale and Keski-Oja, 1997; Romberger, 1997; Lee et al., 2000; Emsley et al., 2000).

Our dtECM preserved essential secreted factors and pathways critical for germ cell proliferation and differentiation, such as chemokine-like receptor (Cmklr2), collagen- and calcium-binding EGF domains (Ccbe1), fibroblast growth factor (FGF), and platelet-derived growth factor (PDGF). Ccbe1 regulates VEGFC signaling, playing a key role in angiogenesis and ECM remodeling during tissue development, inflammation, and wound healing (Arimori et al., 2021; Saldin et al., 2017; Jha, 2014; Roukens et al., 2015). Its calcium- and collagen-binding domains further modulate ECM structure, affecting tissue density and integrity (Beachley et al., 2018; Sasikumar et al., 2019). FGFs are well-known regulators of spermatogonia proliferation and differentiation (Barton et al., 2010; Bos et al., 2011; Jiang et al., 2013; Tian et al., 2019; Hasegawa and Saga, 2014), while PDGFs contribute to gonadal development (Basciani et al., 2010) by influencing Leydig cells, cell proliferation, angiogenesis, and ECM synthesis (Heldin and Westermark, 1999; Betsholtz and Westermark, 2010; Ross et al., 1986; Gnessi et al., 2000; Basciani et al., 2008). Additionally, PD-ECGF supports spermatogonial stem cell renewal in Japanese eel spermatogenesis (Miura et al., 2003; Miura and Miura, 2011) and enhances human spermatogonial stem cell self-renewal in both 2D and 3D culture systems (Khadivi et al., 2020). Likewise, with preserved matrisome components, dtECM is an effective biomaterial for 3D culture (Gharenaz et al., 2020; Baert et al., 2015; Topraggaleh et al., 2019; Vermeulen et al., 2019; Khadivi et al., 2020; Movassagh et al., 2020; Kargar-Abarghouei et al., 2018; Dzobo et al., 2019; Bashiri et al., 2022), enabling testicular organoids and outperforming Matrigel by enhancing SSC proliferation (Baert et al., 2015; Topraggaleh et al., 2019; Yang et al., 2020; Choi et al., 2014; Von Kopylow et al., 2018; Richer et al., 2020; Cortez et al., 2022). By preserving essential growth factors and signaling pathways, our dtECM shows promise for supporting germ cell development and maintaining the spermatogonial stem cell niche, though further *in vitro* studies are needed to assess its functionality.

Furthermore, our decellularization protocol could be adapted for larger, aquaculture-relevant species like rainbow trout (*Oncorhynchus mykiss*), producing fish-dtECM scaffolds with significant biotechnological potential. Available in lyophilized or hydrogel forms, these biomaterials could be used to support 3D spermatogonial stem cell (SSC) cultures, enhancing *in vitro* spermatogenesis, particularly in species with long reproductive cycles (e.g., salmon, sturgeon, tuna, tambaqui, pacu), and offering an innovative complement to cryopreservation. This scalable approach aligns with emerging SSC culture and testicular tissue engineering goals for aquaculture and conservation (Yasui et al., 2022; Shikina and Yoshizaki, 2010; Chen et al., 2022), demonstrating broad applicability across various aquaculture species.

## 5 Conclusion

Overall, our results demonstrate the efficacy of SDS in removing nuclear and cellular components, while preserving essential ECM elements in the dtECM. Importantly, to the best of our knowledge, our study is the first to examine testicular protein composition and provides a comprehensive characterization of retained proteins in fish-derived dtECM. Hence, given that the architecture and the core composition of the dtECM were preserved, the dtECM developed here may provide an optimal 3D culture environment to promote the attachment, proliferation, and differentiation of fish testicular cells. Thus, future studies are warranted to evaluate the *in vitro* performance of these functional scaffolds to supply organoids to support SSC growth.

## Data Availability

The raw data supporting the conclusions of this article will be made available by the authors, without undue reservation.
