# Supplementary material for: Engineering a 3D platform for testis bioengineering: generation and proteomic profiling of decellularized fish testicular scaffolds
Source: Front Bioeng Biotechnol. 2025 Jul 25;13:1631542. doi: 10.3389/fbioe.2025.1631542 (PMC12331664; doi:10.3389/fbioe.2025.1631542)
Supplement: Supplementary file 1 [file DataSheet1.pdf]

Supplementary Material

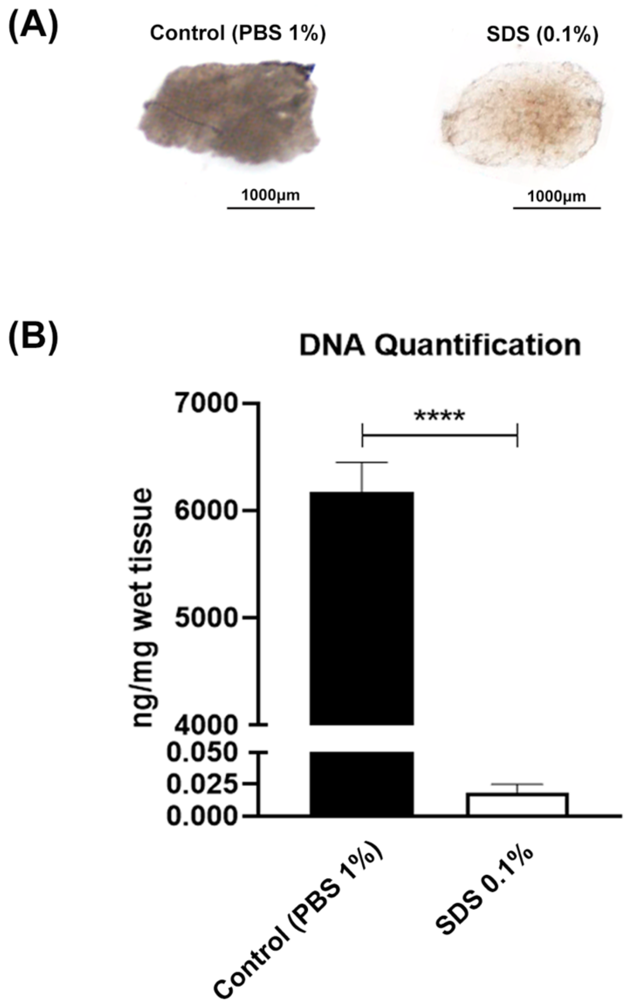

Supplementary Figure 1. Effect of SDS treatment through macroscopic analysis and DNA quantification. (A) Macroscopic images of control (1% PBS) and SDS-treated (0.1%) tissue samples, showing decellularization changes after treatment. Scale bars represent 1000µm. (B) Bar graph representing DNA quantification (ng/mg wet tissue) in control and SDS-treated tissue samples, indicating a significant reduction in DNA content after SDS treatment (**** $p < 0.0001$).

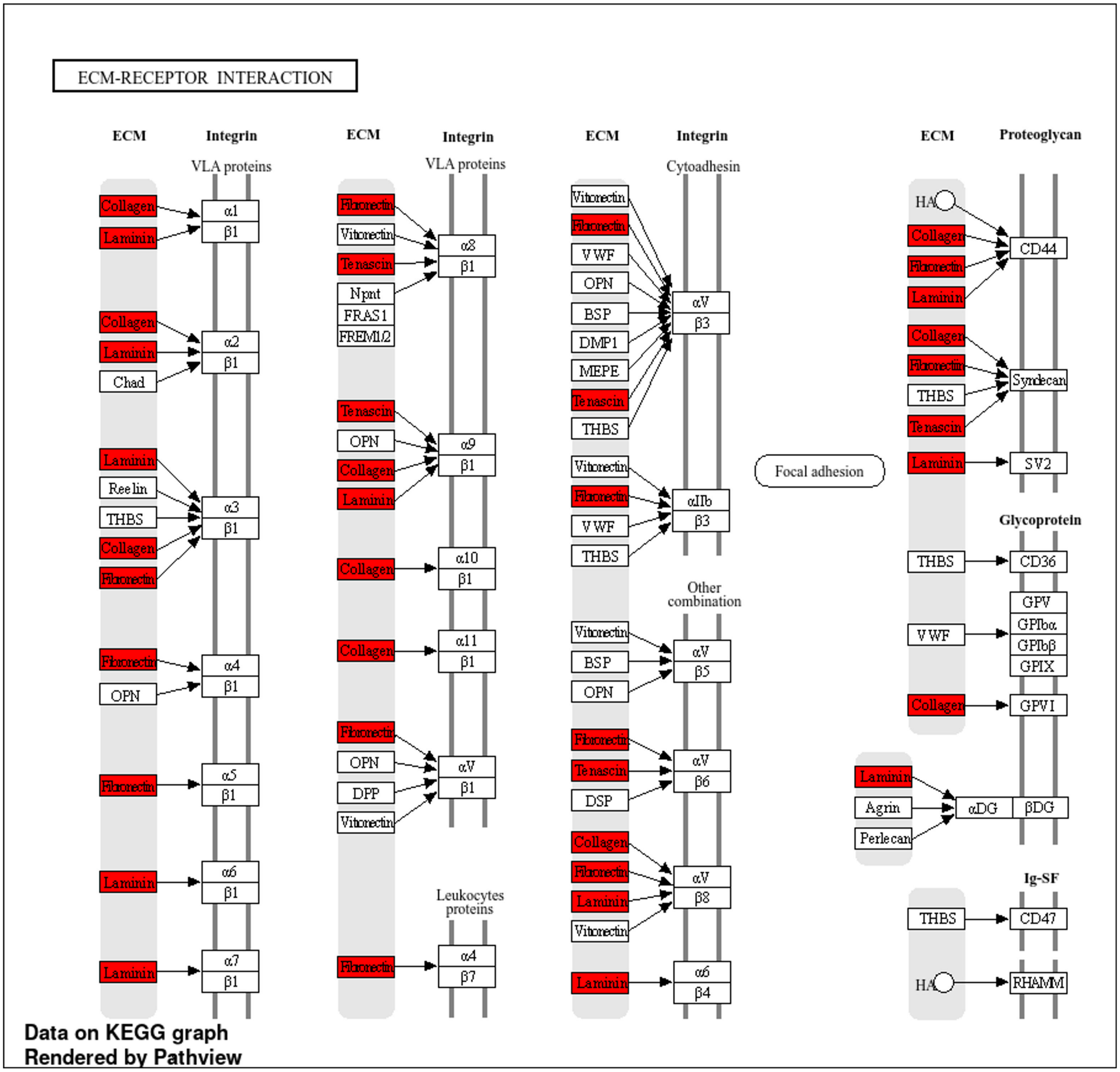

Supplementary Figure 2. The KEGG graph illustrates the ECM-receptor interaction pathway enriched by upregulated (enriched) proteins between native and decellularized testis ECM (dtECM). Uncharacterized orthologs are listed as protein names in supplementary data.

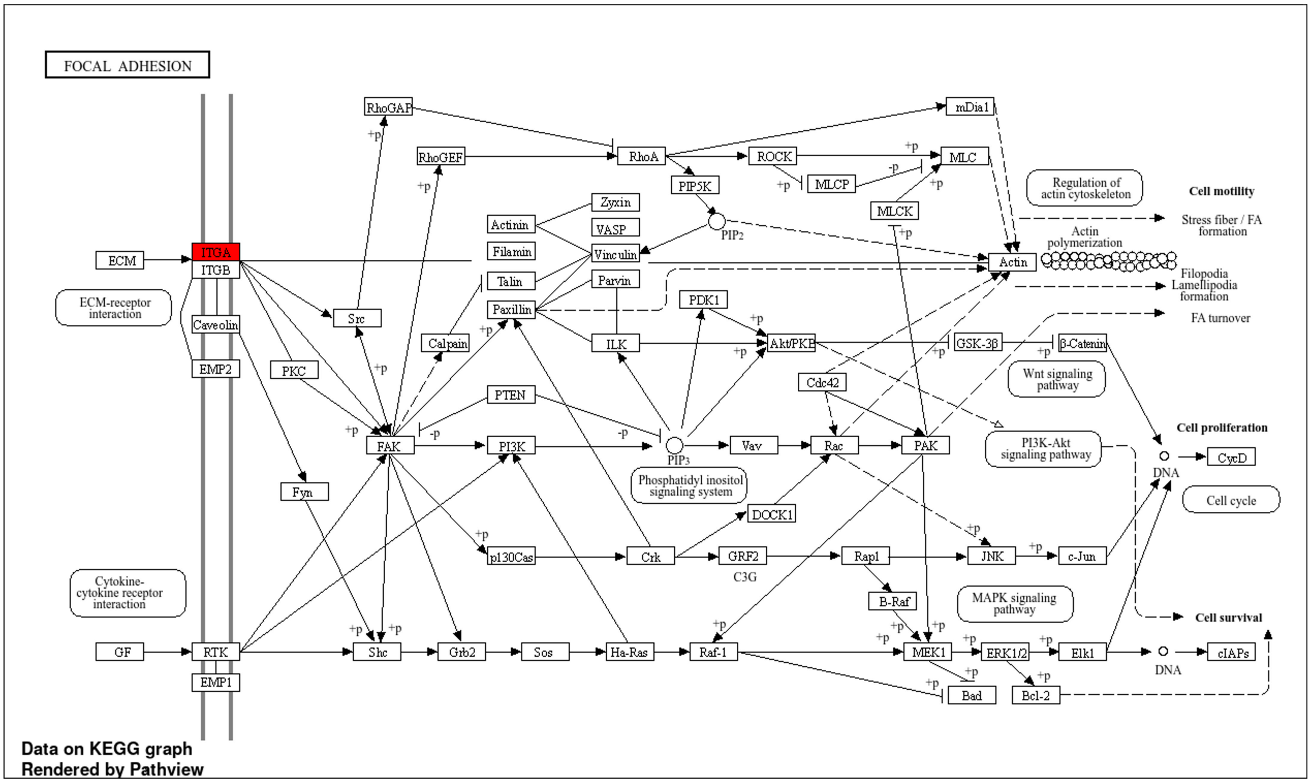

Supplementary Figure 3. The KEGG graph illustrates the focal adhesion pathway enriched by upregulated proteins between native and decellularized testis ECM (dtECM). Uncharacterized orthologs are listed as protein names in supplementary data.

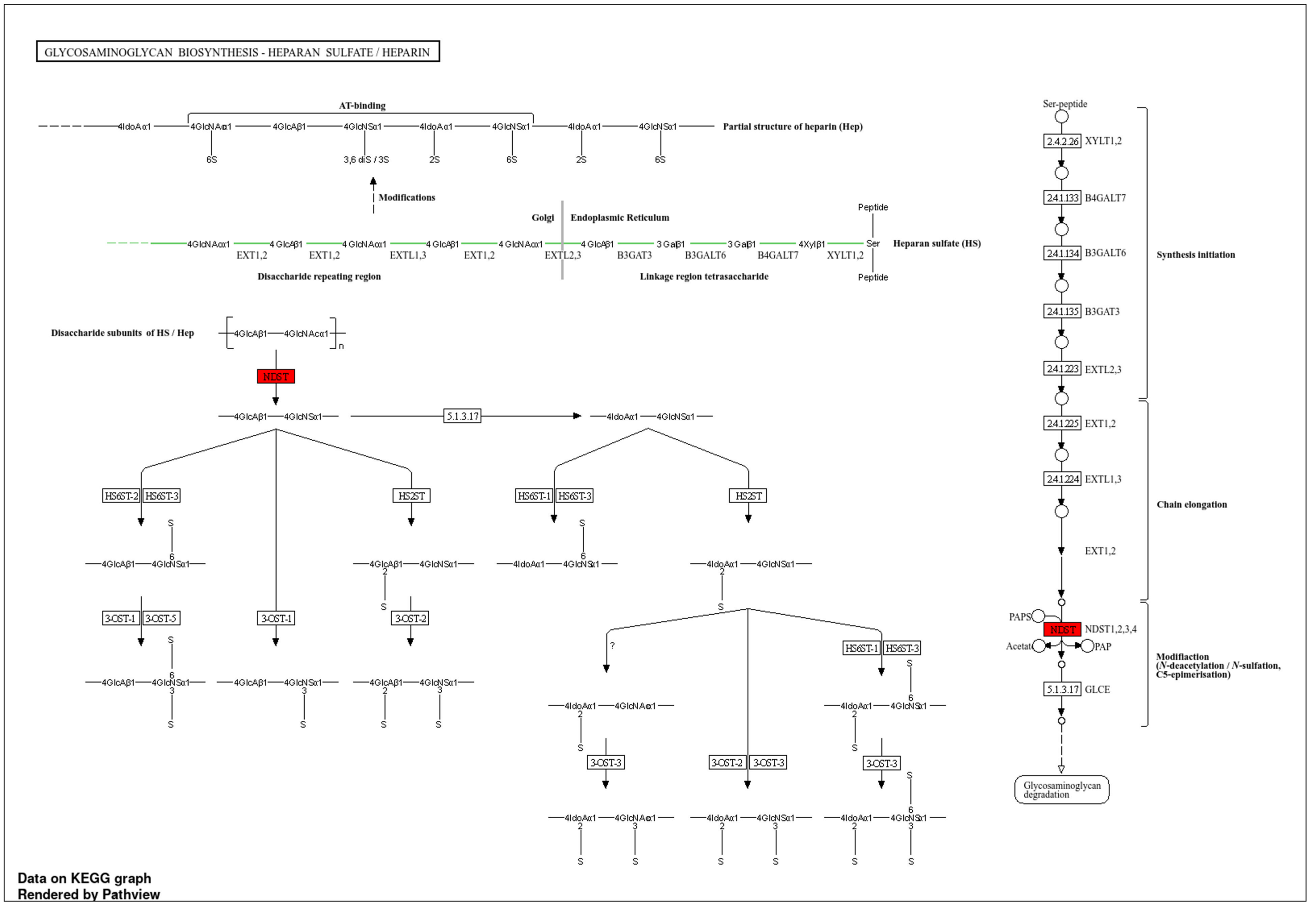

Supplementary Figure 4. The KEGG graph illustrates the glycosaminoglycan biosynthesis (Heparan sulfate/Heparin) pathway enriched by upregulated proteins between native and decellularized testis ECM (dtECM). Uncharacterized orthologs are listed as protein names in supplementary data.

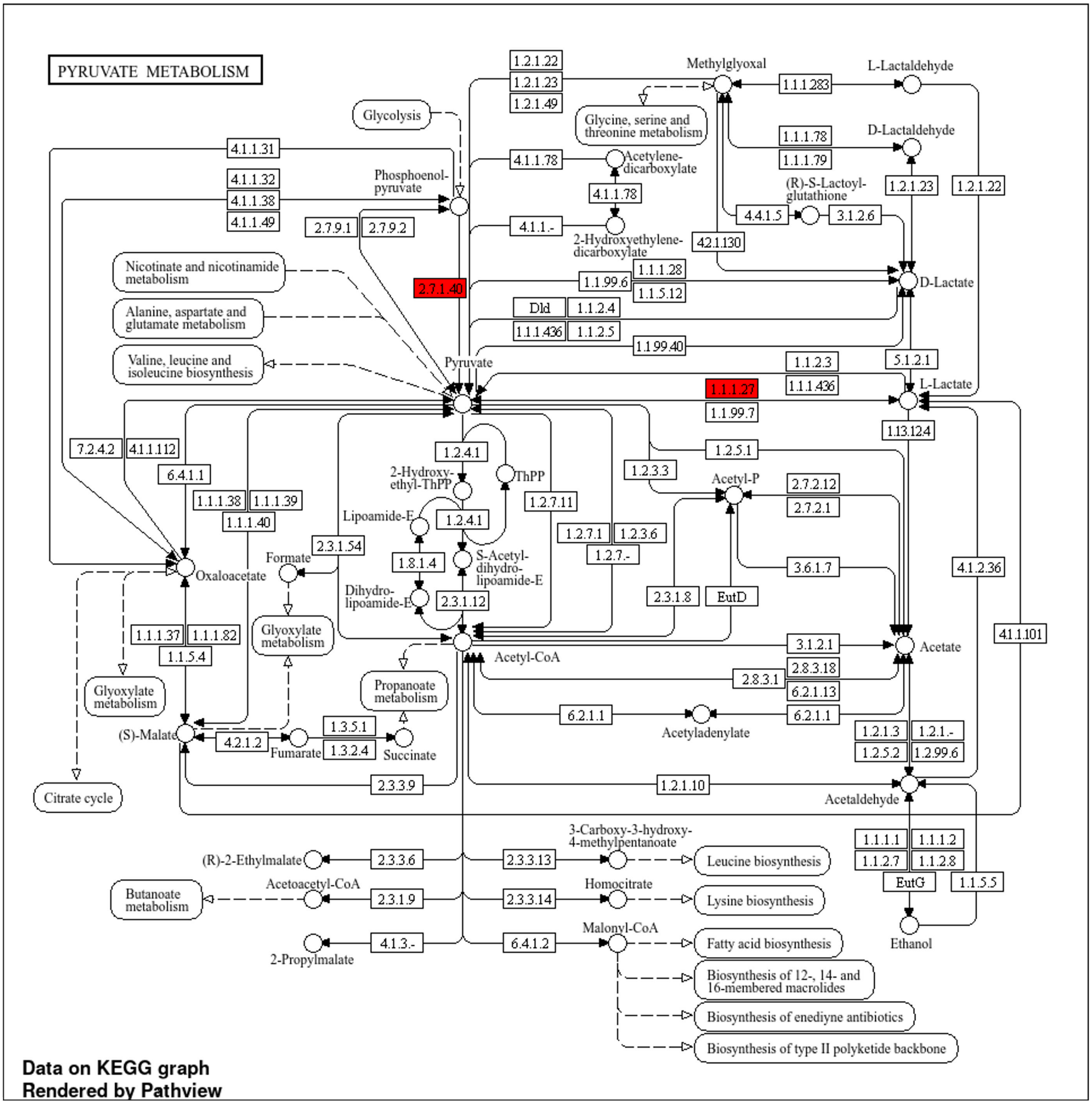

Supplementary Figure 5. The KEGG graph illustrates the pyruvate metabolism pathway enriched by downregulated proteins between native and decellularized testis ECM (dtECM). Uncharacterized orthologs are listed as protein names in supplementary data.

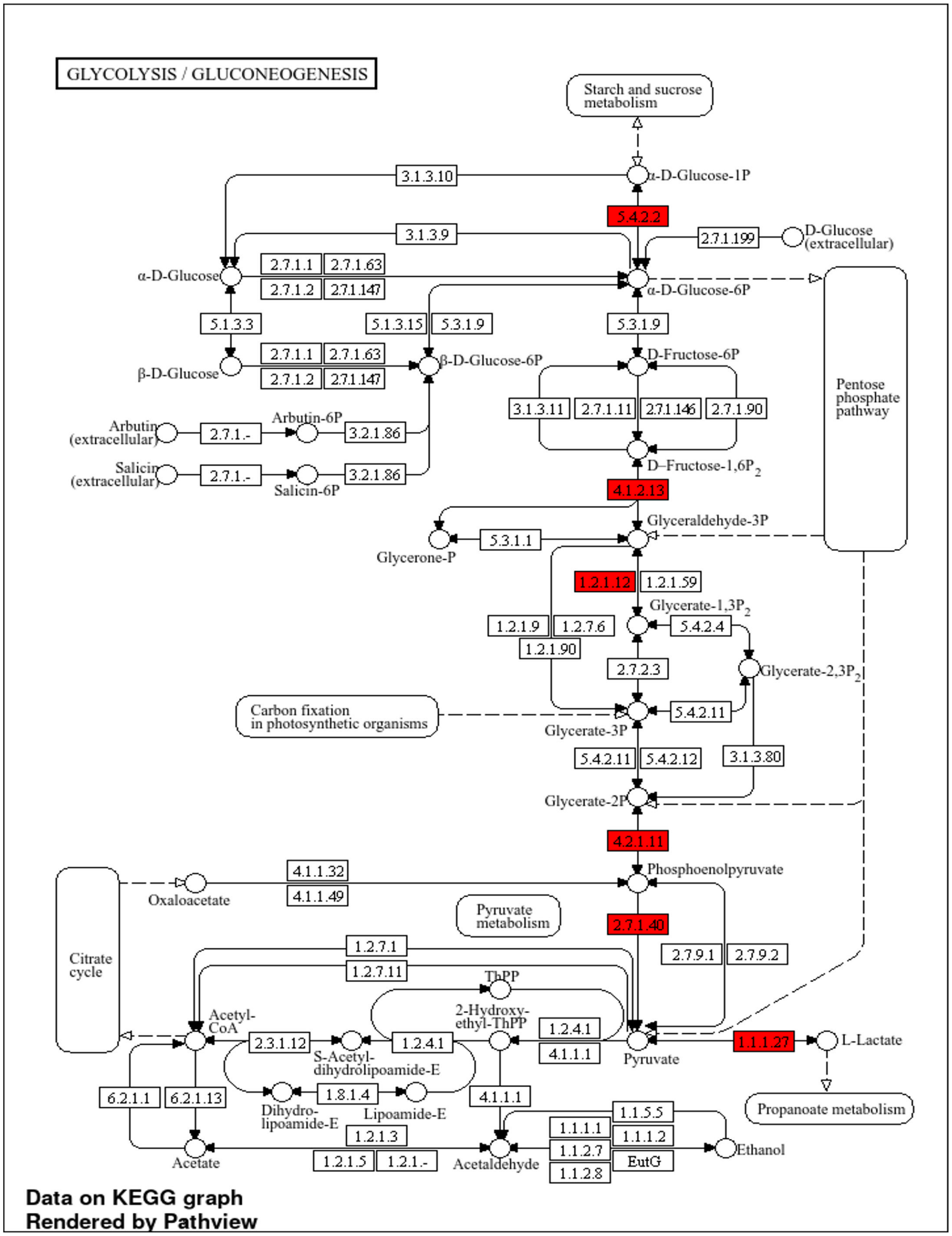

Supplementary Figure 6. The KEGG graph illustrates the glycolysis/Gluconeogenesis pathway enriched by downregulated proteins between native and decellularized testis ECM (dtECM). Uncharacterized orthologs are listed as protein names in supplementary data.

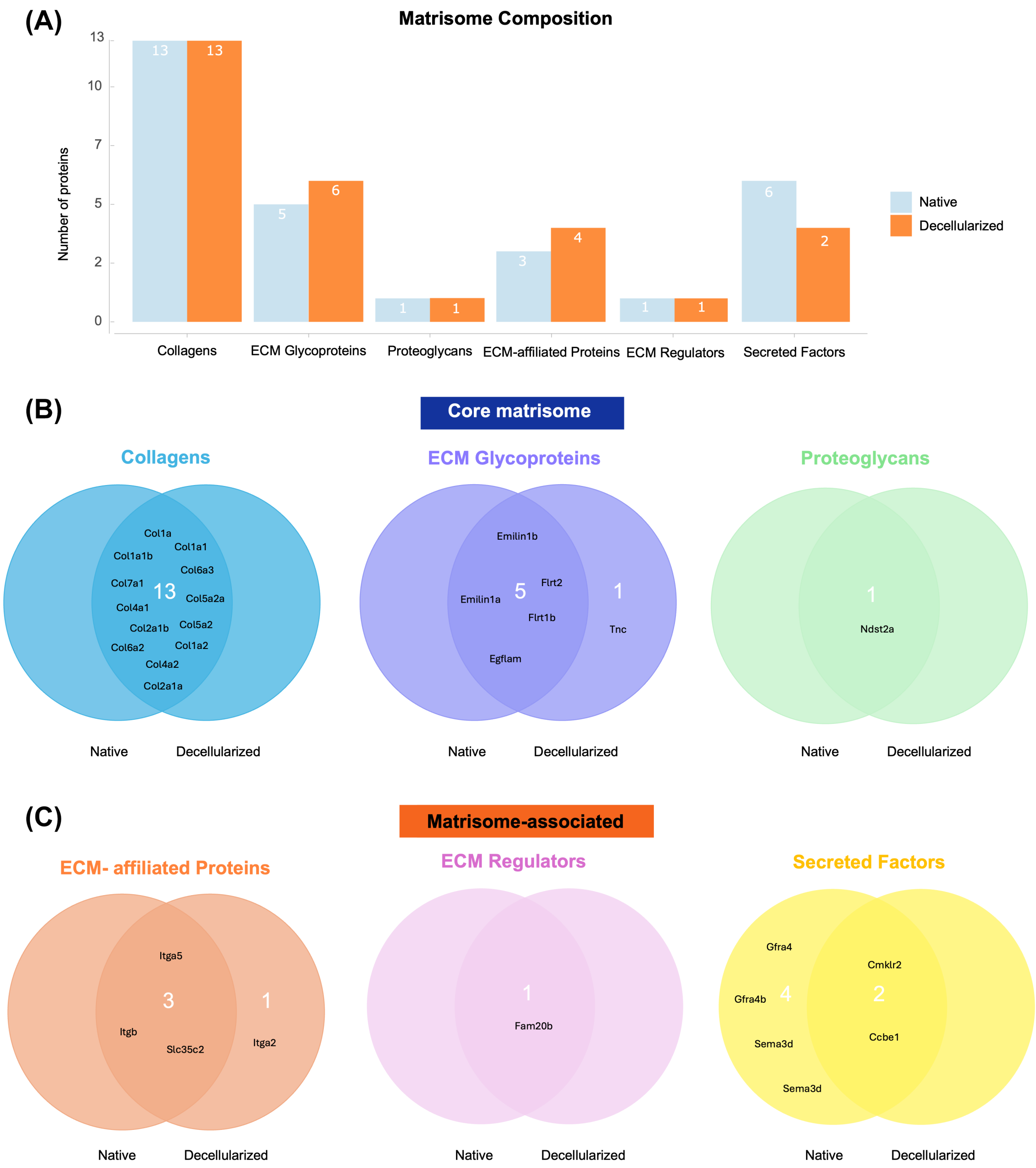

Supplementary Figure 7. Matrisome signature of native and decellularized testis ECM (dtECM). (A) Bar plots represent the number of identified proteins across matrisome subcategories (collagens, ECM glycoproteins, proteoglycans, ECM-affiliated proteins, ECM regulators, and secreted factors) for native and dtECM, highlighting their compositional differences. (B) Venn diagram represents the overlap of Matrisome proteins found in native and decellularized testis ECM (dtECM), as well as the

proteins detected exclusively in each condition. The proteins are grouped based on matrisome divisions and categories.
